# Association between maternal shift work during pregnancy child overweight and metabolic outcomes in early childhood

**DOI:** 10.3389/fpubh.2022.1006332

**Published:** 2022-09-30

**Authors:** Che-Wei Liao, Chih-Fu Wei, Mei-Huei Chen, Wu-Shiun Hsieh, Ching-Chun Lin, Pau-Chung Chen

**Affiliations:** ^1^Institute of Environmental and Occupational Health Sciences, National Taiwan University College of Public Health, Taipei, Taiwan; ^2^Department of Environmental and Occupational Medicine, National Taiwan University College of Medicine and Hospital, Taipei, Taiwan; ^3^Department of Environmental Health, Harvard T.H. Chan School of Public Health, Boston, MA, United States; ^4^Institute of Population Health Sciences, National Health Research Institutes, Miaoli, Taiwan; ^5^Department of Pediatrics, National Taiwan University College of Medicine and Hospital, Taipei, Taiwan; ^6^Department of Pediatrics, Cathay General Hospital, Taipei, Taiwan; ^7^Department of Public Health, National Taiwan University College of Public Health, Taipei, Taiwan; ^8^National Institute of Environmental Health Sciences, National Health Research Institutes, Miaoli, Taiwan

**Keywords:** shift work, overweight, insulin resistance, HOMA-IR, during pregnancy

## Abstract

**Background:**

Previous studies found that maternal shift work during pregnancy was associated with many reproductive hazards, including small for gestational age, preterm birth, stillbirth, and neurodevelopmental impairment. Some studies also showed that these children are more likely to become overweight in early childhood. However, the association with metabolic factors, such as insulin resistance and dyslipidemia, was less studied. Hence, we aimed to understand better the relationship between maternal shift work during pregnancy and the risk of childhood overweight and metabolic outcomes. Confounding factors were also discussed, including diet, exercise, and demographical factors.

**Methods:**

We enrolled pregnant women before delivery in the Taiwan Birth Panel Study (TBPS) II conducted between 2010 and 2012, and followed the children of these participants in 2018. The objective of this study is to investigate the influence of prenatal and postnatal factors on infant and early childhood health. During the follow-up in 2018, we checked children's demographic data, obtained blood specimens, and checked their blood sugar, blood insulin, and lipid profiles. Structured questionnaires were used to evaluate demographic data. Multiple linear and logistic regressions were used to examine the associations between maternal shift work during pregnancy and child overweight, metabolic disorders, such as HOMA-IR, and lipid profiles.

**Results:**

In this study, we included 407 mother-children pairs with different work shifts (350 day workers and 57 shift workers), and a sub-population without underweight children was also created (290 day workers and 47 shift workers). Shift work during pregnancy was associated with a higher Homeostasis Model Assessment-Insulin Resistance index (HOMA-IR) and a higher odds ratio for overweight in children born from mothers doing shift work during pregnancy after adjustment. The findings were attenuated when we investigated the effect of shift work before pregnancy.

**Conclusion:**

Our study suggested that maternal shift work during pregnancy was associated with child overweight and insulin resistance in early childhood.

## Introduction

With the economic growth, there are increasing demands for services around the clock, both business and pleasure/entertainment, thus creating a growing demand for shift work. Although there is no single definition describing the term “shift work” to date, the term “shift work” is generally defined as a considerable proportion of work hours falling outside the daytime working schedule (1, 2). Shift work comprises multiple schedules, including regular evening or night shifts, rotating shifts and irregular shifts, and split shifts (3). The shift workers around the world are not a small portion. In 2010, data from the U.S. National Health Interview Survey showed that ~28.7% of workers are estimated to have alternative shift work (4). Approximately, 20% of all employees work nights in Europe (5). In Taiwan, shift workers increased significantly from 2001 to 2010, from 16.6 to 24% for male workers and from 12 to 20.4% for female workers (6). The high prevalence of shift work has led to concerns about its potential impacts on population health.

Shift work is associated with many problems, such as health and safety effects (7, 8). The safety effects include increased accident rates during working or transportation (9, 10). Given the health effects, previous studies have shown that shift work may alter circadian rhythms (11). Shift work is also associated with increased risks of overweight and obesity (12, 13), type 2 diabetes (13, 14), and cardiovascular diseases (13, 15). Moreover, the International Agency for Research on Cancer has concluded that shift work involving circadian disruption has a probable carcinogenic effect on human breast cancer (13, 16), colorectal cancer (17, 18), and prostate cancer (19).

Maternal shift work is also associated with several reproductive hazards, such as menstrual disruption, early spontaneous pregnancy loss, preterm delivery, low birth weight, small for gestational age (20–22), infant behavior, and neurodevelopment (23). The epidemiological study also showed a relationship between maternal shift work and childhood overweight and obesity (24). In animal studies, rats born from mothers exposed to chronic phase shift, which means three nights with light while a day without light during pregnancy, showed increased adiposity, hyperleptinemia, hyperinsulinemia, and poor glucose in response to a glucose tolerance test (25).

Metabolic syndrome represents heterogeneous metabolic statuses by a cluster of hypertension, dyslipidemia, central obesity, and disturbed glucose control (26). International Diabetes Federation (IDF) published its definition of metabolic syndrome only in adults (27). Meanwhile, metabolic syndrome was not suggested to be diagnosed in children younger than 10 years (28), but insulin sensitivity is inversely correlated with obesity and visceral adiposity in 6–10 years old children (29). Generally, weight is a significant risk factor in the development of metabolic syndrome (30, 31).

Insulin resistance also plays an important pathophysiological role in developing many metabolic abnormalities, especially for the non-diabetes mellitus population in prediction (32), since insulin resistance and insulin secretory dysfunction are precursors of non-insulin-dependent diabetes mellitus, also called type 2 diabetes mellitus (33). The term “insulin resistance” is a pathological condition when cells in the muscles, fat, and liver do not respond well to insulin and cannot easily take up glucose from the blood. It is also a component of metabolic syndrome (34) and a significant risk factor for coronary artery disease (35).

Although plenty of animal studies showed that day-night disturbance induced offspring to become overweight, increasing adiposity, and hyperinsulinemia (25, 36), few epidemiological reports are available on the effect of maternal shift work exposure on children's metabolic outcomes. The gap that is to be filled is whether maternal shift work is associated with biomarkers for childhood metabolic syndrome. Our study hypothesized that maternal shift work might lead to overweight or metabolic disturbances in offspring.

## Materials and methods

### Study population and data collection

The Taiwan Birth Panel Study (TBPS) is a nationwide prospective birth cohort study (37) that aims to investigate the effects of prenatal and postnatal factors on infant and early childhood health in Taiwan. This time, Taiwan Birth Panel Study II (TBPS II) was established for further evaluation of children's health outcomes. A total of 1,030 pregnant women in the second or third trimester were recruited from a tertiary hospital (National Taiwan University Hospital) in Taiwan from 2010 to 2012, of which 18 pregnant women gave birth in other institutions. A structured questionnaire was used to collect the data, including parental demographic factors (e.g., weight and height), socioeconomic factors (e.g., highest education level and family income), health condition (e.g., hypertension and diabetes), substance use (e.g., tobacco, alcohol, and betel nut), dietary intake (e.g., seafood, fruits, and dietary supplements), living environment (e.g., environmental tobacco smoke, incense burning, mold, and cockroach), working condition (e.g., work hours, shift, industrial type, lifting or not, and company size) before and during pregnancy. We also collected data on their offspring, including gestational factors (e.g., parity and mode of delivery) and birth outcomes (e.g., birth weight, height, and gestational age at birth).

Then, we followed 501 mother-children pairs at the National Taiwan University Hospital Children's Hospital in 2018. A structured questionnaire was given to the family at the study site by pre-trained interviewers, which collected updated demographic factors (e.g., weight and height), family income, substance use (e.g., tobacco, alcohol, and betel nut), and also children's data, including demographic factors (e.g., weight and height), health condition (e.g., allergic diseases, hearing, and visual problem), living environment (e.g., environmental tobacco smoke, incense burning, mold, cockroach, and pets), dietary (e.g., meals, snacks, and beverages), exercise (e.g., type, duration, and frequency), and neurobehavior condition (e.g., SNAP-IV questionnaire). During this follow-up, we collected biospecimens, including children's blood, urine, and stool samples, and performed a corresponding physical examination.

### Exposure measurement and definitions

Information on the maternal shift work status before and during pregnancy was obtained from structured questionnaires at the time of recruitment.

We selected four types of work shifts in a priori (day-work, day-work with extended hours to night, night shift, and day-night rotating shift) for the participants. After excluding the non-employed respondents, mothers in the day-work group, which means regular business hours (around 09:00 h to 17:00 h), were categorized as non-shift workers. The other three groups were characterized as shift workers. Although the questionnaires include the shift before and during pregnancy, we classified the participants mainly by their work shift during pregnancy as the main results because we focused on in-uterus effects here.

### Measurement of birth outcome and general health conditions

First, we used birth weight, gender, and gestational age at birth to determine small for gestational age, large for gestational age, or appropriate for gestational age according to the nationwide singleton birth weight percentile by gestational age in Taiwan (38). Specifically, small for gestational age is defined as the birth weight of the newborn below the 10 th percentile for gestational age of the same sex, and large for gestational age means that the birth weight of the newborn is above the 90th percentile for the gestational age of the same sex.

Trained research staff checked children's anthropometric data, including weight, height, waist circumference, and hip circumference. We calculated a child's body mass index (BMI) by their body weight and height according to the formula (weight in kg)/(height in meters)^2^. Then, we use the child and teenage BMI recommendation from Taiwan Health Promotion Administration (HPA) to classify their BMI according to their age and sex. The child would be classified as overweight if BMI was at the 85–95th percentile for specific age and sex. Meanwhile, if the BMI was above the 95th percentile for the corresponding age and sex, the child was classified as obese. Lastly, if BMI was < 5th percentile for specific age and sex, the child would be classified as underweight. Because being underweight is also a health problem, we created a sub-population that excluded the children with BMI < 5th percentile for specific age and sex during the analysis.

### Measurement of metabolic factors

The gold standard of insulin resistance is the clamp technique, including the euglycemic clamp and hyperglycemic clamp technique (39, 40). However, the major limitation of the above techniques includes the time-consuming and invasive procedure, which made it a less practical method for large-scale population studies.

We used the Homeostasis Model Assessment-Insulin Resistance index (HOMA-IR) as a proxy to estimate insulin resistance instead. It employed the product of blood sugar level before meals and blood insulin level (HOMA-IR index = insulin(μU/mL) × glucose(mmol/L)/22.5). A previous study showed a strong correlation between clamp-measured total glucose disposal and HOMA-estimated insulin sensitivity (41). Therefore, HOMA-IR is considered an alternative method for clamp techniques; researchers can reliably use it in large-scale or epidemiological studies in which only a fasting blood sample is available to assess insulin sensitivity (42). The optimal cut-off point for HOMA-IR in the diagnosis of metabolic syndrome, according to a previous study, is considered 2 in males and 2.5 in females (43). We obtained children's blood, and then checked their blood sugar levels and blood insulin before meals. Then the HOMA-IR index was calculated according to the formula.

From their biological samples, we also checked their lipid profile, including total cholesterol (T-CHO), triglyceride (TG), low-density lipoprotein cholesterol (LDL-C), and high-density lipoprotein cholesterol (HDL-C), and compared the levels between the groups. The cut-off points of the abnormal lipid profile of children were based on the established guidelines (44): T-CHO ≥ 200 mg/dL, TG ≥ 100 mg/dL, LDL-C≥ 130 mg/dL, and HDL-C ≤ 40 mg/dL.

### Confounding variables

The confounders can be categorized into two parts: children-related and parent-related factors. Children-related factors include gender (male or female), gestational age (term or preterm), exercise habits, and eating habits. Parent-related factors include parental weight, height, BMI, parity of child (nulliparity or multiparity), mode of delivery (normal spontaneous delivery or cesarean section), maternal age, family income (above or below average), and educational level (junior high school, senior high school, junior college, university, or graduate school level).

Maternal BMI before pregnancy is among the strongest predictors of offspring obesity during early childhood (45). Maternal age also showed a positive association with child overweight (46). Children from multiparous mothers had lower levels of child body mass index, lower levels of total and low-density lipoprotein cholesterol, and a lower risk of child overweight than children of nulliparous mothers (47). A meta-analysis also showed an increased risk of overweight among children born by cesarean section (48).

Low income was associated with a child's overweight or obese status (49). We used the “Investigation report on family income and expenditure in Taipei city” in 2011 and 2018 to evaluate the median family income levels and divided them into two groups by median income level (50, 51).

Secondhand smoking was associated with metabolic disturbance, and previous studies showed lower HDL-C and higher LDL-C, insulin, waist circumference, and body weight in the exposure group (52). Besides, secondhand smoking influences glucose control (53). We obtained information regarding secondhand smoking exposure through questionnaires and incorporated it into our models.

Furthermore, we know that lack of exercise and eating habits are associated with child body weight control, especially snacks and sweet beverages. Therefore, we calculated the child's daily time for exercise, and snack and beverage consumption was also checked according to questionnaires. Candidates for confounding factors included maternal education, maternal BMI before pregnancy, maternal age of birth, family income, child gender, parity, mode of delivery, child exercise time per week, child beverage intake, and secondhand smoke exposure.

### Statistical analysis

The study population was grouped according to maternal work shift during pregnancy. The descriptive characteristics of the study population and underweight exclusive sub-population were presented as the mean and standard deviation for continuous data and as numbers and percentages for categorical data. The chi-square test was applied for the comparison of categorical variables between the two groups, and the Kruskal–Wallis test was applied for continuous variables. Multiple linear regressions were performed to calculate the estimated differences for continuous variables like weight, lipid profiles, and HOMA-IR among different maternal work shift groups. Multiple logistic regressions were performed to calculate the adjusted odds ratio of abnormal BMI, lipid profiles, and HOMA-IR for shift-working mothers. We incorporated confounding factors into our multivariable models by epidemiology evidence. Finally, we picked six variables into models: maternal education, maternal BMI before pregnancy, maternal age of birth, child gender, child exercise time per week, and secondhand smoking exposure. We excluded child beverage intake because diet data was not accurate enough to quantify the intake amount. We asked them about the frequency and amount of beverage intake, but we did not record the type of drinks, as it was hard to calculate total calorie and sugar intake. Another reason is that beverage intake is a possible mediator, not a confounder.

Moreover, we exclude parity and mode of delivery because these factors have multicollinearity with maternal age. Family income was also excluded because it may have multicollinearity with maternal education level.

We also used multiple linear and logistic regressions, adjusted for the same confounders above, to compare the differences in biomarkers and the adjusted odds ratio of variables between maternal day work and shift work before pregnancy. We hypothesized that the effect of shift work before pregnancy is less than the shift work during pregnancy, and the results are presented in Supplementary Tables 1, 2.

We compared the baseline characteristics of included children and parents between different body mass index trajectories of children, and the results are presented in Supplementary Tables 3, 4. We presented parents' educational and occupational details between two work shift groups in Supplementary Table 5 and the comparison of beverage intake between two groups in Supplementary Table 6.

We demonstrated the result in Supplementary Table 7 to examine the differences in demographic characteristics between followed and non-followed subjects. A two-tailed test of < 0.05 is defined as statistical significance throughout the study.

All analyses mentioned above were conducted with SAS software version 9.4.

## Results

### Baseline characteristics of the population

In this study, 1,030 mother-infant pairs were enrolled from 2010 to 2012, and 1,012 children were born at the National Taiwan University Hospital; among them, 501 received follow-up in 2018. Then, we further analyzed the population with complete work shift data during pregnancy. First, we excluded 94 subjects (83 non-employed mothers and 11 without work shift data), which resulted in a study population including 407 subjects. Among these 83 participants who were not working while pregnant, 53 of the 83 mothers were not working before their pregnancy. Meanwhile, there were 364 day-working and 84 shift-working mothers before pregnancy, and 350 day-work and 57 shift-work mothers during pregnancy after excluding 11 mothers with incomplete data. The mother-child pairs were further assigned to our study population.

**Figure 1:**
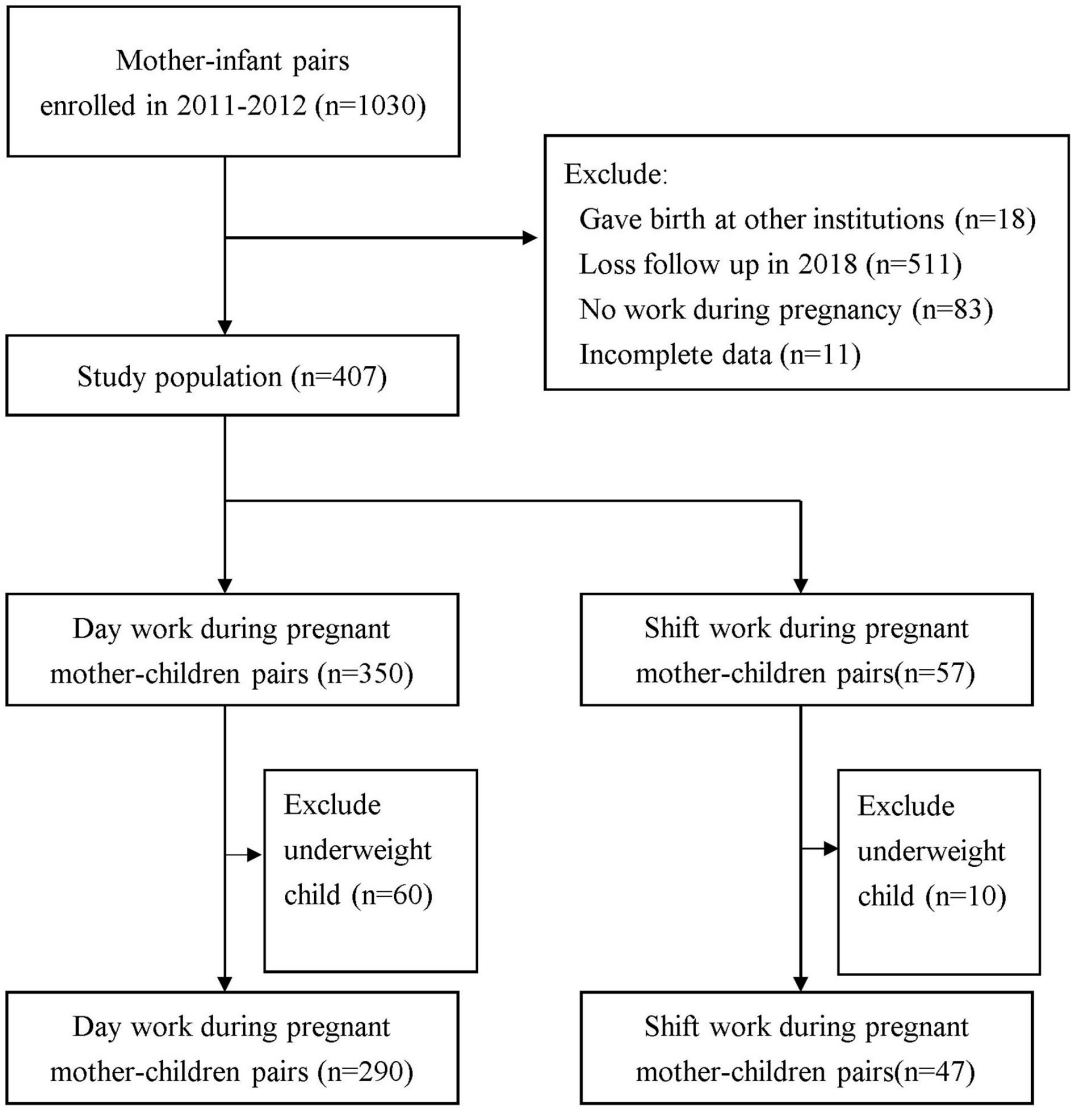
Flowchart of study population selection.

Meanwhile, a subgroup excluding underweight children, defined as the children with BMI < 5th percentile for specific age and sex, was also included, which comprised 290 day-work and 47 shift-work mother-child pairs. Figure 1 shows the flowchart of the selection process of the study groups.

The demographic characteristics of the study population are listed in Table 1. We used the chi-square and non-parametric tests to compare the characteristics among different shift groups for the total population and sub-population model without underweight children. The exercise time per week, beverage, and snack intake of children were not different between the two groups, and the distributions were not substantially altered when we excluded underweight children.

**Table 1:** Baseline characteristics of children and their parents.

	**Total population**			**Sub-population (Exclude underweight children)**		
	**Maternal day work group** **(*n =* 350)**	**Maternal shift-work group (*n =* 57)**	***p*-value ^b^**	**Maternal day work group** **(*n =* 290)**	**Maternal shift-work group** **(*n =* 47)**	**p-value**
**Parent factor**						
Maternal age of pregnancy	33.6 ± 3.5^a^	33.2 ± 4.0	0.519	33.6 ± 3.5	32.9 ± 3.9	0.267
Maternal height (cm)	159.4 ± 7.1	158.4 ± 14.1	0.721	159.3 ± 7.5	158.2 ± 15.4	0.894
Maternal weight before pregnancy (kg)	54.4 ± 8.5	54.1 ± 8.9	0.955	54.9 ±8.8	54.5 ± 8.7	0.846
Maternal weight during pregnancy (kg)^c^	66.4 ± 9.1	67.6 ± 10.7	0.164	67.0 ± 9.5	68.0 ± 11.0	0.3
Gestational weight gain (kg)	11.8 ± 7.6	14.5 ± 12.7	*0.042	11.8 ± 8.2	14.6 ± 13.9	0.146
Maternal BMI before pregnant	21.6 ± 6.7	23.1 ± 15.0	0.981	21.9 ± 7.3	23.7 ± 16.5	0.841
Maternal BMI during pregnant	26.4 ± 8.2	29.0 ± 20.9	0.182	26.7 ± 8.9	29.7 ± 22.9	0.359
Paternal height (cm)	173.0 ± 5.6	172.3 ± 4.9	0.535	172.6 ± 5.4	171.9 ± 4.6	0.52
Paternal weight (kg)	76.5 ± 13.0	76.3 ± 11.3	0.996	77.0 ± 13.6	77.0 ± 11.3	0.865
Paternal BMI	25.5 ± 3.7	25.7 ± 3.5	0.691	25.7 ± 3.8	26.0 ± 3.5	0.572
Family high income level (%)	86 (24.6)	15 (26.3)	0.778	75 (25.9)	11 (23.4)	0.721
**Occupational factor**						
Maternal industry types			0.169			0.056
Professional service^d^	156 (44.6)	31 (55.4)		129 (44.5)	28 (59.6)	
Others	194 (55.4)	26 (45.6)		161 (55.5)	19 (40.4)	
**Child behavior factor**						
Beverage (serving/day)	0.2 ± 0.2	0.2 ± 0.2	0.977	0.2 ± 0.2	0.2 ± 0.2	0.927
Snack (serving/day)	0.7 ± 0.5	0.8 ± 0.4	0.198	0.8 ± 0.5	0.8 ± 0.4	0.268
Exercise (hours/week)	2.2 ± 3.4	2.3 ± 3.2	0.874	2.1 ± 3.0	2.3 ± 3.2	0.66
Secondhand smoke exposure	32 (9.1)	9 (15.8)	0.124	27 (9.3)	8 (17.0)	0.11

^a^Data were are presented with as the mean ± standard deviation or n (%).

^b^P- value is the result of the Wilcoxon test (non-parameter statistics).

^c^Mother's weight during pregnancyt is recorded from the latest routine prenatal visit.

^d^Professional service includes education, science research, medical, pharmacist, lawyer, architect, accountant, journalist, escrow officer, religion worker, publishing, and information science.

* means *p* < 0.05.

Regarding parental factors, we compared maternal age, maternal and paternal height, maternal weight before pregnancy and before delivery, maternal gestational weight gain, paternal weight, parental body mass index, and parental highest education level. The above-mentioned demographic characteristics were recorded in 2010–2012 when the child was ready to be delivered, and there was no significant difference between the two groups. The result was almost the same after we excluded underweight children. We recorded family income levels in 2010 and 2018, and the results showed no significant difference between the two groups.

Regarding occupational factors, we compared the maternal industry types, maternal company size (workers above 30 or not), and lifting work or not. Lifting work occurred more frequently in the shift-work group, while the industry type and company size showed no differences between the two groups. If we excluded the underweight children, the result was similar. However, more than 25% of the participants had missing data on the question “lifting work or not” (107 among 407 subjects), which could lead to non-differential bias toward the null result.

### Health outcome of the children at birth and after 7 years

The health outcomes of the study population at birth and after 7 years are listed in Table 2. For the total participants, the male gender of children seemed to be a little higher than the female gender in the shift work group but did not reach statistical significance. If we excluded the underweight children, the male gender of children was even higher, but still did not reach statistical significance.

**Table 2:** Birth and 7-year-old health outcomes of children.

	**Total population**			**Sub-population (Not include underweight children)**		
	**Maternal day work group** **(*n =* 350)**	**Maternal shift-work group** **(*n =* 57)**	***p*-value ^b^**	**Maternal day work group** **(*n =* 290)**	**Maternal shift-work group** **(*n =* 47)**	***p*-value**
**Birth outcome**						
Male (%)	188 (53.7)^a^	36 (63.2)	0.185	149 (51.4)	31 (66)	0.065
Gestational age (weeks)	38.5 ± 2.8	38.8 ± 1.7	0.294	38.7 ± 1.7	38.8 ± 1.8	0.536
Mode of delivery (NSD)	222 (63.7)	39 (68.4)	0.493	181 (65.2)	34 (72.3)	0.337
Parity	210 (60.0)	36 (63.2)	0.589	171 (59.0)	30 (63.8)	0.484
Birth Height (cm)	48.9 ± 2.8	49.5 ± 2.4	0.054	49.1 ± 2.6	49.6 ± 2.4	0.073
Birth Weight (gram)	3,076.0 ± 530.9	3,170.9 ± 526.8	0.19	3,126.2 ± 497.1	3,188.3 ± 540.0	0.355
Small for gestational age	45 (12.9)	6 (10.5)	0.623	38 (13.1)	4 (8.5)	0.378
Large for gestational age	33 (9.4)	7 (12.3)	0.504	31 (10.7)	6 (12.8)	0.674
**Seven-year-old health outcome**						
Age (years)	7.4 ± 0.7	7.3 ± 0.6	0.632	7.3 ± 0.7	7.4 ± 0.6	0.682
Height (cm)	124.3 ± 6.7	124.7 ± 6.9	0.691	124.4 ± 6.8	125.7 ± 6.6	0.226
Weight (kg)	23.9 ± 4.9	25.1 ± 6.6	0.237	24.7 ± 5.0	26.4 ± 6.5	0.058
Body mass index (BMI)	15.4 ± 2.2	15.9 ± 2.7	0.173	15.9 ± 2.2	16.5 ± 2.6	0.075
Waist circumference	53.5 ± 6.8	55.0 ± 7.2	0.123	54.3 ± 6.9	56.5 ± 7.1	*0.028
Waist-hip ratio (WHR)	0.85 ± 0.07	0.86 ± 0.04	0.125	0.85 ± 0.07	0.86 ± 0.04	0.089
HOMA-IR^d^	1.16 ± 1.47	1.61 ± 2.35	0.521	1.25 ± 1.58	1.81 ± 2.54	0.403
HOMA-IR abnormal	27 (7.7)	8 (14.0)	0.116	25 (8.6)	8 (17.0)	0.074
Triglyceride (TG)	61.3 ± 25.6	63.2 ± 31.2	0.402	61.9 ±26.8	67.4 ± 32.7	0.769
TG abnormal^c^	21 (6.0)	7 (12.3)	0.084	20 (6.9)	7 (14.9)	0.063
LDL-C	94.4 ± 23.9	88.6 ± 22.5	0.123	94.3 ± 23.4	91.8 ± 22.6	0.705
LDL-C abnormal	31 (8.9)	3 (5.3)	0.365	24 (8.3)	3 (6.4)	0.66
HDL-C	64.9 ± 14.1	62.0 ± 13.1	0.136	64.5 ± 13.8	60.5 ± 12.5	0.059
HDL-C abnormal	7 (2.0)	2 (3.5)	0.475	7 (2.4)	2 (4.3)	0.471

^a^Data are presented as the mean ± standard deviation or n (%).

^b^P-value is the result of the Wilcoxon test (non-parameter statistics).

^c^Abnormal lipid profile cut-off: TG ≥ 100 mg/dL, LDL ≥ 130 mg/dL, and HDL < 40 mg/dL.

^d^The cut-off is 2 in males and 2.5 in females.

HOMA-IR, Homeostasis Model Assessment-Insulin Resistance; NSD, normal spontaneous delivery; LDL-C, low-density lipoprotein cholesterol; HDL-C, high-density lipoprotein cholesterol.

As for birth outcomes, the gestational age, parity, mode of delivery, birth weight, birth height, and proportion of small for gestational age and large for gestational age were not different between the two groups. If we excluded underweight children, the result was similar.

Seven-year-old health outcomes, the weight, body mass index, waist circumference, and waist-hip ratio of children were marginally higher in the shift work group by the mean value. Meanwhile, body height was similar between the two groups. If we excluded underweight children, the waist circumference reached statistical significance (mean of shift workers childre*n* = 56.5 cm and mean of day workers children = 54.3 cm, *p* = 0.028). Meanwhile, the shift-work group had a higher mean value of body height, weight, body mass index, waist circumference, and waist-hip ratio of children.

About biomarkers, we checked TG, LDL-C, HDL-C, blood insulin, pre-prandial blood sugar, and the Homeostasis Model Assessment-Insulin Resistance index (HOMA-IR). There was a marginally higher average of HOMA-IR and TG among the shift work group, while LDL-C and HDL-C levels were lower in the shift-work group. If we calculated the abnormal rate, the abnormal rate for HOMA-IR, TG, and HDL-C was higher. In contrast, the LDL-C abnormal rate was non-significantly lower in the shift-work group. If we excluded underweight children, the results showed a similar trend. HOMA-IR and TG were higher, while LDL-C and HDL-C levels were lower in the shift-work group. The abnormal HOMA-IR, TG, and HDL-C were higher, while the LDL-C abnormal rate was lower in the shift-work group. But they did not reach statistical significance, whether underweight children were included or not.

### Linear and logistic regression of children's birth outcomes

In order to check the effect of birth outcome influenced by maternal shift-work during pregnancy after adjusting for confounding factors, we used multiple linear and logistic regression analyses, and the results are summarized in Table 3. We estimated the differences for each parameter between mothers exposed to shift work during pregnancy vs. their day-working references. We showed the unadjusted odds ratio (OR) for each outcome between shift work during pregnancy group and their day-working reference group. The result showed no significant difference in birth weight, birth height, small for gestational age, and large for gestational age.

**Table 3:** Linear and logistic regression of birth outcome in children.

	**Unadjusted**				**Adjusted** ^**c**^			
	***β^a^***	**SE**		***p*-value**	***β^a^***	**SE**		***p*-value**
Birth weight (gram)	83.8	76.4		0.273	94.9	75.6		0.209
Birth height (cm)	0.6	0.4		0.133	0.6	0.4		0.115
	**OR** ^b^	**95% CI**		***P*** **-value**	**OR**	**95% CI**		***P*** **-value**
Small for gestational age	0.80	0.32	1.97	0.623	0.79	0.32	2.00	0.625
Large for gestational age	1.35	0.57	3.21	0.503	0.95	0.35	2.63	0.927

^a^β is the estimated difference between mothers exposed to shift work during pregnancy vs. their day-working references for each parameter, before and after adjustment for other covariates.

^b^OR is the unadjusted odds ratio for each outcome between shift work during pregnancy and their reference group (day working during pregnancy).

^c^The results demonstrate estimates comparing shift-working mothers and day-working mothers, and models were adjusted for maternal BMI before pregnancy, maternal age of birth, child gender, and child secondhand smoke exposure.

cm, centimeter; CI, confidence interval; OR, odds ratio; SE, standard error.

Then, we presented the results in models adjusted for the confounding variables, including maternal BMI before pregnancy, maternal age of birth, child gender, and secondhand smoke exposure. The result showed no significant difference in birth weight, birth height, small for gestational age, and large for gestational age after adjustment.

### Maternal shift work during pregnancy and child health outcome at 7-year-old follow-up, linear regression

We summarized the results of the multiple linear regression for the continuous health outcomes in Table 4. The estimated difference between mothers exposed to shift work during pregnancy and their day-working references was compared with beta-estimates. All of the health outcomes are 7-year-old child health outcomes. In the total population, the beta-estimates of weight, body mass index, waist circumference, waist-hip ratio, TG, and HOMA-IR were higher among the shift work exposed group, and the HOMA-IR among mothers doing shift work during pregnancy was marginally higher (β = 0.45, SE = 0.23, *p* = 0.054). The β-estimates of LDL-C and HDL-C were negative, without statistical significance. The β-estimate of weight was significantly higher in the sub-population excluding underweight children (β = 1.71, SE = 0.81, *p* = 0.035). The β-estimate of HOMA-IR also showed statistical significance (β = 0.55, SE = 0.27, *p* = 0.044).

**Table 4:** Linear regression for associations between maternal shift work during pregnancy and health outcome of children at 7-year-old follow-up.

		**Unadjusted**			**Adjusted model** ^**d**^		
		***β^a^***	**SE**	**p**	***β^a^***	**SE**	**p**
Weight (kg)	T^b^	1.18	0.74	0.111	0.72	0.73	0.324
	S^c^	1.71	0.81	*0.035	1.16	0.82	0.156
Body mass index (BMI)	T	0.56	0.33	0.088	0.40	0.33	0.215
	S	0.68	0.35	0.053	0.50	0.35	0.156
Waist circumference	T	1.56	0.97	0.109	1.20	0.97	0.218
	S	2.10	1.09	0.055	1.72	1.11	0.121
Waist-hip ratio (WHR)	T	0.01	0.01	0.301	0.01	0.01	0.364
	S	0.012	0.01	0.267	0.01	0.01	0.301
Triglyceride (TG)	T	1.89	3.77	0.616	1.57	3.77	0.678
	S	5.45	4.34	0.208	5.41	4.38	0.217
Low-density lipoprotein (LDL-C)	T	−5.75	3.38	0.089	−5.26	3.33	0.114
	S	−2.5	3.66	0.495	−1.16	3.60	0.748
High-density lipoprotein (HDL-C)	T	−2.91	1.99	0.143	−2.31	2.00	0.246
	S	−3.96	2.14	0.065	−3.16	2.19	0.149
Homeostatic Model Assessment for Insulin Resistance (HOMA-IR)	T	0.45	0.23	0.054	0.44	0.22	*0.049
	S	0.55	0.27	*0.044	0.56	0.27	*0.038

^a^β is the estimated difference between mothers exposed to shift work during pregnancy vs. their day-working references for each parameter, before and after adjusting for other covariates.

^b^T means total population (N = 407), which includes 350 children delivered by day workers, and 57 children delivered by shift workers.

^c^S means Sub-population, which excluded underweight children (N = 70), leaving 290 children of day workers and 47 children of shift workers.

^d^The results demonstrate estimates comparing shift-working mothers and day-working mothers, and models were adjusted for maternal education, maternal BMI before pregnancy, maternal age of birth, child gender, child exercise, and child secondhand smoking exposure.

SE, standard error. * means *p* < 0.05.

Then, we conducted multivariable regression, adjusting for maternal education, maternal BMI before pregnancy, maternal age of birth, child gender, child exercise time per week, and secondhand smoke exposure during pregnancy. In the total population of the adjusted model, β-estimates of weight, body mass index, waist circumference, waist-hip ratio, and TG were positive, while LDL-C and HDL-C levels were negative. HOMA-IR reached the statistical significance in the total population (β = 0.44, SE = 0.22, *p* = 0.049) and the sub-population (β = 0.56, SE = 0.27, *p* = 0.038).

### Maternal shift work during pregnancy and child health outcome at 7-year-old follow-up, logistic regression

We set the abnormal cut-off of BMI according to the Child and Teenage BMI recommendation from the Taiwan Health Promotion Administration and the TG, HDL-C, and LDL-C levels according to the Expert Panel on Integrated Guidelines for Cardiovascular Health and Risk Reduction in Children and Adolescents (44). Then, multiple logistic regression analyses were used to analyze the risk between different work shifts, and the results are presented in Table 5.

**Table 5:** Logistic regression for associations between maternal shift work during pregnancy and health outcome of children at 7-year-old follow-up.

		**Unadjusted**				**Adjusted Model** ^**d**^			
		**OR^a^**	**95% CI**		***P*-value**	**OR**	**95% CI**		***P*-value**
Overweight	T^b^	2.46	1.16	5.23	*0.019	2.33	1.05	5.18	*0.038
	S^c^	2.55	1.18	5.52	*0.017	2.46	1.08	5.63	*0.033
Abnormal BMI^e^	T	1.66	0.92	2.99	0.091	1.60	0.87	2.94	0.129
HOMA-IR abnormal^f^	T	1.95	0.84	4.54	0.120	2.22	0.92	5.36	0.077
	S	2.17	0.92	5.16	0.078	2.46	0.99	6.11	0.052
TG abnormal^g^,	T	2.19	0.89	5.43	0.089	2.31	0.89	6.03	0.086
	S	2.36	0.94	5.90	0.068	2.55	0.95	6.84	0.062
LDL-C abnormal^g^	T	0.57	0.17	1.94	0.369	0.64	0.18	2.23	0.480
	S	0.76	0.22	2.62	0.659	0.94	0.26	3.41	0.925
HDL-C abnormal^g^	T	1.78	0.36	8.80	0.478	1.39	0.23	7.34	0.700
	S	1.80	0.36	8.92	0.474	1.37	0.26	7.32	0.710

^a^OR is the unadjusted odds ratio for each outcome between shift work during pregnancy and their reference group (day-working during pregnancy).

^b^T means total population (N = 407), which includes 350 children delivered by day workers, and 57 children delivered by shift workers.

^c^S means Sub-population, which excluded underweight children (N = 70), leaving 290 children of day workers and 47 children of shift workers.

^d^The results demonstrate estimates comparing shift-working mothers and day-working mothers, and the model was adjusted for maternal education, maternal BMI before pregnancy, maternal age of birth, child gender, child exercise, and child secondhand smoking exposure.

^e^We referred to Taiwan Health Promotion Administration (HPA) for the national age-dependent cut-off for abnormal BMI, including overweight/obesity and underweight.

^f^The cut-off is 2 in males and 2.5 in females.

^g^Abnormal lipid profile cut-off: TG ≥ 100 mg/dL, LDL-C ≥ 130 mg/dL, and HDL-C < 40 mg/dL.

TG, triglyceride; LDL-C, low-density lipoprotein cholesterol; HDL-C, high-density lipoprotein cholesterol. * means *p* < 0.05.

The unadjusted odds ratio for each outcome is estimated between shift work during pregnancy and their reference (day-working) group. Information regarding all health outcomes was collected from the children upon their follow-up. The OR of overweight was significantly higher in the shift-work group in both total and sub-population (OR = 2.46, 95% CI: 1.16–5.23, *p* = 0.019 in total population; OR = 2.55, 95% CI: 1.18–5.52, *p* = 0.017 in sub-population), while OR of abnormal weight (including overweight, obesity, and underweight), TG, LDL-C, HDL-C, and HOMA-IR showed no significant difference.

Then, we adjusted the variables, including maternal education, maternal BMI before pregnancy, maternal age of birth, child gender, child exercise time per week, and secondhand smoking exposure. In both total and sub-population, the adjusted OR of overweight was significantly higher in the shift-work group (aOR = 2.33, 95% CI: 1.05–5.18, *p* = 0.038 in total population; aOR = 2.46, 95% CI: 1.08–5.63, *p* = 0.033 in sub-population). In the sub-population, abnormal HOMA-IR risk was marginally higher in the shift-work group (aOR = 2.46, 95% CI: 0.99–6.11, *p* = 0.052).

### Sensitivity analysis: Maternal shift work before pregnancy and child health outcome

To estimate the effect of maternal shift work before pregnancy, we selected the population by shift before pregnancy on the questionnaires, where 364 day workers and 84 shift workers before pregnancy were chosen. We analyzed the health effect of children on their maternal shift work during pregnancy using the same set of confounding variables, and a sub-population was created by excluding underweight children. Then, we adjusted for maternal education, maternal BMI before pregnancy, maternal age of birth, child gender, child exercise time per week, and secondhand smoke exposure as in the main analysis.

Multiple linear regression analyses were performed, and the results are summarized in Supplementary Table 1. No significant difference in parameters was noted in weight, BMI, and HOMA-IR between the two groups. But HDL-C was significantly lower in the children of the shift-work group with and without adjustment (unadjusted model: β = −3.85, SE = 1.65, *p* = 0.020; adjusted model: β = −3.53, SE = 1.65, *p* = 0.033). In multiple logistic regression analyses, the results are summarized in Supplementary Table 2. The odds ratio of overweight and HOMA-IR were not significantly higher, but in the adjusted model, aOR of abnormal TG was higher in the shift-work group (total population: aOR = 2.53, 95% CI: 1.05–6.12, *p* = 0.039; sub-population: aOR = 2.67, 95% CI: 1.08–6.60, *p* = 0.034).

### Sensitivity analysis for other covariates

We compared demographic characteristics between “children with normal or underweight” and “children with overweight or obesity” to examine whether the confounding variables reflected known associations or not. The results are presented in Supplementary Table 3. Maternal weight and BMI before pregnancy, maternal weight during pregnancy, paternal weight and BMI, and birth weight were significantly higher in children with overweight or obesity. In contrast, maternal highest education level and family income level were significantly higher in the children with normal weight or underweight. Besides, exercise hours per week for children were marginally higher in children with normal or underweight (*p* = 0.050). The results showed that the parental body size was highly associated with their child; parent income and education level also played crucial roles in child body shape in early childhood. Our result showed that exercise time was crucial to maintaining body weight. Lastly, beverage and snack intake was non-significantly higher in children with overweight or obesity.

We also compared the differences between overweight, normal, and underweight children, as presented in Supplementary Table 4. Children's age was higher, while gestational age, birth weight, and birth height were lower in the underweight group. Regarding parental factors, maternal weight before and during pregnancy, paternal weight, and BMI showed the same trend as children's body size; the underweight children group had lower parental body weights, while children with overweight had higher parental body weights. Maternal educational level was higher in the underweight group. Children's exercise time was longer in the underweight group and shortest in the overweight group.

We also compared other parental factors like maternal hometown, maternal smoking and drinking during pregnancy, parental educational level in detail, and maternal industrial types. Maternal “lifting work or not” showed a difference, but more than 25% of participants had missing data (107 among 407 subjects). As for other factors, no significant differences were observed between the two groups. Similar results were observed if we excluded underweight children. The above results are presented in Supplementary Table 5.

We also listed beverage intake details in Supplementary Table 6. According to questionnaires, we calculated the frequency and the amount of beverage intake to compare the beverage intake between the two groups, and no significant difference was found between them.

To ensure the population followed up in 2018 was similar to the loss follow-up population, we compared parental demographic data and birth outcomes between the groups with no significant difference between them, and the results are presented in Supplementary Table 7.

### Sensitivity analysis for regression models: Model fit and collinearity

We used the Hosmer–Lemeshow test to examine our model's goodness-of-fit. The goodness-of-fit test results passed the Hosmer–Lemeshow test in the adjusted model in total population and sub-population.

We also checked the Examination of the Correlation Matrix to see if any of the variables included have a high correlation and no high correlation between variables. We also checked the variance inflation factor (VIF) and tolerance for multicollinearity. In all models, no tolerance values fell below 0.1, and no variance inflation factor was above the value of 10. It indicated that multicollinearity was not likely.

## Discussion

In our study, we found that the waist circumference of the shift-work group children was higher than the day-work group in the sub-population when we excluded underweight children, and the body weight of the shift-work group children was marginally higher than their non-shift counterparts. The shift-work group children may have higher waist circumference before adjusting for confounding factors. We then used multiple linear and logistic regression to analyze the hypothesis that maternal shift work during pregnancy is associated with childhood overweight and metabolic disturbance. In the multiple linear regression model, the children in the shift-work group showed higher body weight in the unadjusted model when we excluded underweight children. However, in the adjusted model, no significant difference was noted. In the multiple logistic regression model, the odds ratio of child overweight was significantly higher in the shift-work group than in the day-work group; a similar result was noted after we excluded underweight children. After we adjusted for confounding factors, the adjusted odds ratio of overweight children was also higher in the shift-work group. A previous study also showed similar results (24).

We also found that, in the multiple linear regression model, HDL-C was lower in the children of the shift-work group but without significance. HDL-C is the so-called “good cholesterol”, which could prevent cardiovascular risk at a high level. A previous study showed an association between shift work and lipid disorder (54, 55), but a few studies investigated the maternal shift work relationship with child lipid disorder. In the multiple logistic regression model, no significant difference between the groups was observed; the reason may be low abnormal subjects (< 5%), and most children were within the normal range.

We also checked insulin sensitivity. In the multiple linear regression model, higher HOMA-IR was seen in the children of the shift-work group after adjustment; the result was more prominent when we excluded underweight children. In the multiple logistic regression model, the adjusted odds ratio of abnormal HOMA-IR was also higher in the shift-work group but did not reach statistical significance. The above results support our hypothesis that maternal shift work during pregnancy may be associated with metabolic disturbances, such as insulin resistance or dyslipidemia.

We expected that shift work during pregnancy may disrupt offspring metabolic programming. The effect in the uterus and placenta would be higher than before pregnancy (56), so we assume that shift work during pregnancy would impose a higher risk for children than shift work before pregnancy. As expected, beta-estimates of HOMA-IR value and odds ratio of overweight were attenuated in shift work before pregnancy. However, if we compared the effect of shift work before pregnancy, HDL-C was even lower in the shift-work group “before” rather than “during” pregnancy. A similar finding in TG was also noted. In the multiple logistic regression model, abnormal TG risk was higher in the shift-work “before” pregnancy group, but no such finding in the shift-work “during” pregnancy group was observed. We assume that some reason other than shift work contributed to the abnormal lipid profile, and we thought the small case number was a possible reason. Regarding HDL-C levels, six children of day workers and three children of shift workers before pregnancy met the cut-off of low HDL-C, while seven children of day workers and two children of shift workers during pregnancy met the cut-off. Regarding TG levels, 20 children of day workers and 9 children of shift workers before pregnancy met the cut-off of high TG, while 21 children of day workers and 7 children of shift workers before pregnancy met the cut-off of high TG.

We made a subgroup without underweight children because the body mass index of < 5th percentile for specific age and sex may be linked to some health problems. Metabolic abnormality is not uncommon among the underweight population, who are also at risk of cardiovascular disease (57). Being underweight due to malnutrition increases the mortality risk among children (58, 59). For the above reasons, we proposed underweight as another abnormal body weight status that could not be classified as normal body size. It is a possible confounder influencing our results, so we thought it necessary to create this subgroup. As for our hypothesis, we found abnormal body weight and HOMA-IR in the shift-work subgroup without underweight children.

Regarding other occupational factors, we found that the mother in the shift-work group was more likely to do lifting work. According to a previous study, heavy lifting would lead to preterm delivery (PTD), low birth weight, and small for gestational age (22, 60). However, we did not put this factor into the adjusted model because there was limited evidence for an association between childhood overweight and lifting work during pregnancy, and the data were missing for over 1/4 of the study population.

The possible reasons as to why maternal shift-work during pregnancy is linked to offspring overweight and insulin resistance are listed below. One is lack of family support, as some studies showed that long working hours and shift-work of mothers are associated with an elevated risk of childhood overweight (61). The higher risk is that they cannot prepare healthy foods for their children and do physical activity with them (62, 63). Another reason is physical inactivity and restrained food choices among the shift workers themselves and an elevated risk for future metabolic diseases, which further influence their offspring (63).

There are two putative biological mechanisms for the associations identified. One is vitamin D3 insufficiency because shift workers may stay in a dark environment longer than day workers in the daytime, while vitamin D3 is related to sunlight exposure (64). Some studies showed that vitamin D3 deficiency is related to visceral adiposity, metabolic syndrome, cancers, and autoimmune, psychiatric, and neurodegenerative diseases, but the causative role of vitamin D3 deficiency in many of these conditions remains unclear (65). As some studies reported, we hypothesized vitamin D3 levels might be lower in shift workers than in day workers (66, 67). However, it remains controversial, as some researchers suggest shift workers should not have vitamin D3 deficiency, partly because some foods have been fortified with vitamin D3, such as milk and orange juice, while some foods, such as eggs and salmons, contain natural vitamin D3 (68, 69).

The other condition is melatonin disturbance, and the author thought the mechanism is probably related to melatonin based on animal studies (25, 70). An animal study showed that circadian clock disruptions would affect maternal behavior and melatonin levels (70). Melatonin is a hormone secreted by the pineal gland, acts as an internal chrono-biotic agent, and synchronizes organismal physiology and behavior between day and night (71). Besides, melatonin has insulin-like hypoglycemic, anabolic, and anti-cholesterol effects (72). Melatonin also modulates adrenal cortisol production and lipolysis in brown adipose tissue (73). Maternal pineal melatonin production increases during pregnancy; circulating melatonin crosses the placenta and reaches the fetal circulation, and then influences fetal development and programming (74). At the same time, melatonin receptors have been widespread in the embryo and fetus since the early stages of pineal gland development (75). A previous study also showed that maternal plasma melatonin rhythm drives the fetal circadian system, for example, changing some rhythms of fetal corticosterone levels (76). In some models, melatonin affects reproductive function and is essential in fetal development and metabolic programming (77). For day workers, the melatonin level is regular, consistent, and stable, with a peak at around 0–2 a.m.; for night workers, the melatonin level is irregular, with no constant pattern and random peak level time. On average, the peak melatonin levels for night workers are lower than for day workers, which occur at around 4–8 p.m. (78).

The strength of our study is a prospective cohort design; we followed subjects for 7–8 years before completing this article. Moreover, we obtained biomarkers, including blood glucose, insulin, and lipid profiles, to confirm our hypothesis with consistency across different markers. Besides, comprehensive covariates are collected, including children-related factors (gender, gestational age, exercise habits, eating habits, and secondhand smoking exposure) and parent-related factors (parental weight, height, BMI, parity of child, mode of delivery, maternal age, family income, and educational level). Thus, we adjusted for possible confounding variables in regression models.

However, several limitations must be acknowledged in this study. First, our population was selected from a single tertiary hospital in the urban area, and generalizability might be a possible concern. However, in previous studies, the proportion of shift workers in our study was similar to the 12–20.4% average in Taiwan (6). Second, the sample size was small, and the 95% confidence intervals were wide. Besides, the result may also be influenced by extreme values. Third, the accuracy of snack or sweet beverage intake is doubted. In the dietary questionnaire, we calculated the frequency of snacks or sweet beverages (at least once a month, once a week, or once a day) times the amount (0, below 1 cup a day, or 1–3 or more cups a day). We could not get the information if the cup size was similar for every participant, and the type of drink may have different sugar content and calories. It was hard to quantify the total beverage amount. Besides, the total calorie intake was crucial for weight gain, but we could not record that information in detail. Fourth, we did not know the maternal shift status after birth, which may lead to different parenting styles that could influence their child's body weight. Lastly, we did not have maternal blood melatonin or vitamin D3 levels during pregnancy, and hence we cannot confirm that the maternal melatonin disturbance or vitamin D3 deficiency hypothesis is valid for the time being.

## Conclusion

Maternal shift work during pregnancy was associated with increased or overweight or obesity in children between 7 and 8 years old after multivariable adjustment.

Furthermore, the Homeostasis Model Assessment-Insulin Resistance index (HOMA-IR) was also higher in both unadjusted and adjusted models, indicating higher insulin resistance. Although there was no evident difference in lipid profile when comparing shift work status during pregnancy, the high-density lipoprotein cholesterol (HDL-C) level was significantly lower, and the triglyceride level was higher in the mother doing shift work before pregnancy.

Finally, our study showed that maternal shift-work during pregnancy might be associated with overweight and insulin resistance, in early childhood, at around 7 years of age. We suggest further study to investigate the biological mechanism and future risk for metabolic diseases.

## Data availability statement

The original contributions presented in the study are included in the article/Supplementary material, further inquiries can be directed to the corresponding author/s.

## Ethics statement

The studies involving human participants were reviewed and approved by National Taiwan University Hospital Research Ethics Committee (201702038RINA). Written informed consent to participate in this study was provided by the participants' legal guardian/next of kin.

## Author contributions

M-HC conceived the project. W-SH, C-CL, and P-CC supervised the project. C-WL and C-FW performed and interpreted statistical analysis results and drafted the manuscript. All authors read and approved the final manuscript.

## Funding

This study was supported by the Ministry of Science and Technology (MOST-106-3114-B-002-002, MOST-107-2321-B-002-028, MOST-109-2621-M-002-020, MOST-110-2621-M-002-022, and MOST-111-2621-M-002-022). The funders had no role in study design, data collection and analysis, decision to publish, or preparation of the manuscript.

## Conflict of interest

The authors declare that the research was conducted in the absence of any commercial or financial relationships that could be construed as a potential conflict of interest.

## Publisher's note

All claims expressed in this article are solely those of the authors and do not necessarily represent those of their affiliated organizations, or those of the publisher, the editors and the reviewers. Any product that may be evaluated in this article, or claim that may be made by its manufacturer, is not guaranteed or endorsed by the publisher.
