# Supplementary material for: Association between maternal shift work during pregnancy child overweight and metabolic outcomes in early childhood
Source: Front Public Health. 2022 Sep 30;10:1006332. doi: 10.3389/fpubh.2022.1006332 (PMC9565036; doi:10.3389/fpubh.2022.1006332)
Supplement: Supplementary file 1 [file Table_1.docx]

| **Supplementary Table S1.**  **Linear regression for associations between maternal shift work before pregnancy and health outcome for children at seven-year-old follow-up** | | | | | | | | | | | |
| --- | --- | --- | --- | --- | --- | --- | --- | --- | --- | --- | --- |
|  |  |  | Unadjusted | | |  |  | Adjusted Model ^d^ | | |  |
|  |  |  | *β*^a^ | SE | p |  |  | *β*^a^ | SE | p |  |
| Weight (kg) | T^b^ |  | 1.04 | 0.65 | 0.107 |  |  | 0.76 | 0.64 | 0.237 |  |
|  | S^c^ |  | 1.36 | 0.70 | 0.054 |  |  | 0.99 | 0.71 | 0.161 |  |
| Body mass index (BMI) | T |  | 0.44 | 0.28 | 0.120 |  |  | 0.34 | 0.28 | 0.224 |  |
|  | S |  | 0.46 | 0.30 | 0.121 |  |  | 0.32 | 0.30 | 0.279 |  |
| Waist circumference | T |  | 1.15 | 0.84 | 0.168 |  |  | 0.98 | 0.84 | 0.239 |  |
|  | S |  | 1.38 | 0.93 | 0.138 |  |  | 1.16 | 0.94 | 0.218 |  |
| Waist-hip ratio (WHR) | T |  | 0.01 | 0.01 | 0.560 |  |  | 0.00 | 0.01 | 0.599 |  |
|  | S |  | 0.01 | 0.01 | 0.485 |  |  | 0.01 | 0.01 | 0.652 |  |
| Triglyceride (TG) | T |  | 3.07 | 3.05 | 0.314 |  |  | 3.26 | 3.03 | 0.281 |  |
|  | S |  | 4.65 | 3.46 | 0.180 |  |  | 5.18 | 3.46 | 0.134 |  |
| Low density lipoprotein (LDL-C) | T |  | -1.99 | 2.84 | 0.485 |  |  | -1.23 | 2.79 | 0.659 |  |
|  | S |  | -0.11 | 3.00 | 0.971 |  |  | 1.70 | 2.97 | 0.568 |  |
| High density lipoprotein (HDL-C) | T |  | -3.85 | 1.65 | *0.020 |  |  | -3.53 | 1.65 | *0.033 |  |
|  | S |  | -3.40 | 1.77 | 0.055 |  |  | -2.96 | 1.80 | 0.099 |  |
| HOMA-IR | T |  | 0.22 | 0.20 | 0.269 |  |  | 0.23 | 0.19 | 0.235 |  |
|  | S |  | 0.26 | 0.23 | 0.264 |  |  | 0.27 | 0.23 | 0.234 |  |
| 1. β is the estimated difference between mothers exposed to shift-work before pregnancy versus their day working references for each parameter, before and after adjusted for other covariates. | | | | | | | | | | | |
| 1. T means total population (N=448), which includes children delivered by day-workers (N=364), and children of shift-workers (N=84) | | | | | | | | | | | |
| 1. S means Sub-population, which excluded underweight children (N=76) , leaving 301 children of day-workers and 71 children of shift-workers | | | | | | | | | | | |
| 1. The results demonstrate estimates comparing shift working mothers and day-working mothers, and model was adjusted for maternal education, maternal BMI before pregnancy, maternal age of birth, child gender, child exercise, child secondhand smoking exposure. | | | | | | | | | | | |
