# Supplementary material for: Association between maternal shift work during pregnancy child overweight and metabolic outcomes in early childhood
Source: Front Public Health. 2022 Sep 30;10:1006332. doi: 10.3389/fpubh.2022.1006332 (PMC9565036; doi:10.3389/fpubh.2022.1006332)
Supplement: Supplementary file 2 [file Table_2.docx]

| **Supplementary Table S2. Logistic regression for associations between maternal shift work before pregnancy and health outcome for children at 7-year-old follow-up** | | | | | | | | | | | | | |
| --- | --- | --- | --- | --- | --- | --- | --- | --- | --- | --- | --- | --- | --- |
|  | |  | Unadjusted | | | |  |  | Adjusted Model ^d^ | | | |  |
|  |  |  | OR^a^ | 95% CI | | P-value |  |  | OR | 95% CI | | P-value |  |
| Overweight | T^b^ |  | 1.67 | 0.84 | 3.31 | 0.143 |  |  | 1.58 | 0.77 | 3.25 | 0.215 |  |
|  | S^c^ |  | 1.65 | 0.82 | 3.31 | 0.158 |  |  | 1.51 | 0.72 | 3.15 | 0.276 |  |
| Abnormal BMI ^e^ | T |  | 1.20 | 0.72 | 2.01 | 0.490 |  |  | 1.16 | 0.68 | 1.98 | 0.584 |  |
| HOMA-IR abnormal ^f^ | T |  | 1.46 | 0.71 | 3.02 | 0.305 |  |  | 1.63 | 0.76 | 3.50 | 0.207 |  |
|  | S |  | 1.60 | 0.76 | 3.36 | 0.217 |  |  | 1.73 | 0.79 | 3.77 | 0.169 |  |
| TG abnormal ^g^ | T |  | 2.06 | 0.90 | 4.70 | 0.085 |  |  | 2.53 | 1.05 | 6.12 | *0.039 |  |
|  | S |  | 2.16 | 0.93 | 4.99 | 0.073 |  |  | 2.67 | 1.08 | 6.60 | *0.034 |  |
| LDL-C abnormal | T |  | 0.86 | 0.35 | 2.13 | 0.739 |  |  | 0.96 | 0.38 | 2.44 | 0.929 |  |
|  | S |  | 0.87 | 0.32 | 2.38 | 0.793 |  |  | 1.07 | 0.38 | 3.03 | 0.899 |  |
| HDL-C abnormal | T |  | 2.21 | 0.54 | 9.00 | 0.269 |  |  | 1.81 | 0.43 | 7.63 | 0.419 |  |
|  | S |  | 2.17 | 0.53 | 8.90 | 0.282 |  |  | 1.80 | 0.43 | 7.63 | 0.423 |  |
| 1. OR is the unadjusted odds ratio for each outcome between shift-work during pregnancy to their reference group day-working during pregnancy | | | | | | | | | | | | | |
| 1. T means total population (N=448), which includes children delivered by day-workers (N=364), and children of shift-workers (N=84) | | | | | | | | | | | | | |
| 1. S means Sub-population, which excluded underweight children (N=76) , leaving 301 children of day-workers and 71 children of shift-workers | | | | | | | | | | | | | |
| 1. The results demonstrate estimates comparing shift working mothers and day-working mothers, and model was adjusted for maternal education, maternal BMI before pregnancy, maternal age of birth, child gender, child exercise, child secondhand smoking exposure | | | | | | | | | | | | | |
| 1. We referred to Taiwan HPA for age-dependent cut-off for abnormal BMI, including overweight/obesity and underweight. | | | | | | | | | | | | | |
| 1. The cutoff is 2 in male and 2.5 in female | | | | | | | | | | | | | |
| 1. Abnormal Lipid profile cutoff: TG ≥ 100 mg/dL, LDL-C ≥ 130 mg/dL, HDL-C < 40 mg/dL | | | | | | | | | | | | | |
| Abbreviation: TG, triglyceride ; LDL-C, low-density lipoprotein cholesterol ; HDL-C, high-density lipoprotein cholesterol. | | | | | | | | | | | | | |
