# Supplementary material for: Association between maternal shift work during pregnancy child overweight and metabolic outcomes in early childhood
Source: Front Public Health. 2022 Sep 30;10:1006332. doi: 10.3389/fpubh.2022.1006332 (PMC9565036; doi:10.3389/fpubh.2022.1006332)
Supplement: Supplementary file 3 [file Table_3.docx]

| **Supplementary Table S3.** **Baseline characteristics of all children with follow-up at 7-years-old between different BMI groups** **(2 groups)** | | | |
| --- | --- | --- | --- |
|  | Children with normal or underweight (n=444) | Children with overweight  (n=57) | p-value |
| Age | 7.4 ± 0.7^a^ | 7.2 ± 0.6 | 0.184 |
| Male gender | 232 (52.3) | 36 (63.2) | 0.185 |
| Gestational age (weeks) | 38.4 ± 3.2 | 38.7 ± 1.6 | 0.281 |
| Mode of delivery (NSD) ^b^ | 288 (64.9) | 38 (66.7) | 0.789 |
| First born | 262 (59) | 31 (54.4) | 0.371 |
| Birth Height(cm) | 48.9 ± 2.7 | 49.5 ± 2.3 | 0.112 |
| Birth Weight(gram) | 3079.7 ± 527.8 | 3238.2 ± 476.2 | 0.032 |
| Maternal age | 33.4 ± 3.6 | 33.5 ± 4.4 | 0.849 |
| Maternal height(cm) | 159.4 ± 8.2 | 160.4 ± 5.7 | 0.268 |
| Maternal weight before pregnant(kg) | 53.9 ± 8.0 | 59.5 ± 11.0 | *<0.001 |
| Maternal weight during pregnant(kg)^c^ | 66.1 ± 8.8 | 71.7 ± 11.8 | *0.001 |
|  |  |  |  |
| Gestational weight gain(kg) | 12.1 ± 6.3 | 12 ± 15.5 | 0.950 |
| Maternal BMI before pregnant | 21.6 ± 8.0 | 23.2 ± 4.2 | *0.017 |
| Maternal BMI during pregnant | 26.5 ± 10.4 | 27.9 ± 4.2 | 0.062 |
| Paternal height(cm) | 172.7 ± 5.5 | 173.8 ± 5 | 0.155 |
| Paternal weight | 75.3 ± 12.2 | 82 ± 13.2 | *<.0001 |
| Paternal BMI | 25.2 ± 3.5 | 27.4 ± 3.7 | *<.0001 |
| Beverage (serving/day) | 0.15 ± 0.2 | 0.2 ± 0.3 | 0.387 |
| Snack (serving/day) | 0.73 ±0.5 | 0.81 ± 0.4 | 0.285 |
| Exercise (hours/week) | 2.2 ± 3.3 | 1.6 ± 2.1 | 0.0501 |
| Maternal high education level (%) | 358 (80.6) | 38 (66.7) | *0.037 |
| Paternal high education level (%) | 361 (81.3) | 41 (71.9) | 0.095 |
| Family high income level (%) | 111 (25.0) | 7 (12.3) | *0.01 |
| 1. Data were presented with the mean ± standard deviation or n (%) 2. Maternal weight during pregnant is recorded from the latest routine prenatal visit | | | |
| Abbreviation: BMI, body mass index ; NSD, normal spontaneous delivery. | | | |
