# Supplementary material for: Association between maternal shift work during pregnancy child overweight and metabolic outcomes in early childhood
Source: Front Public Health. 2022 Sep 30;10:1006332. doi: 10.3389/fpubh.2022.1006332 (PMC9565036; doi:10.3389/fpubh.2022.1006332)
Supplement: Supplementary file 4 [file Table_4.docx]

| **Supplementary Table S4. Baseline characteristics of all children with follow-up at 7-years-old between different BMI groups (3 groups)** | | | | |
| --- | --- | --- | --- | --- |
|  | Underweight children (n=82) | Normal weight children (n=362) | Overweight children (n=57) | p-value |
| Age | 7.5 ± 0.6 ^a^ | 7.3 ± 0.6 | 7.2 ± 0.6 | 0.043 |
| Male (%) | 50 (61) | 182 (50.3) | 36 (63.2) | 0.064 |
| Gestational age (weeks) | 37.6 ± 4.7 | 38.6 ± 2.7 | 38.7 ± 1.6 | 0.031 |
| Mode of delivery (NSD ) | 47 (57.3) | 241 (66.6) | 38 (66.7) | 0.275 |
| First born | 52 (63.4) | 210 (58) | 31 (54.4) | 0.377 |
| Birth Height(cm) | 48.3 ± 3.4 | 49.1 ± 2.6 | 49.5 ± 2.3 | 0.013 |
| Birth Weight(g) | 2894.1 ± 576.7 | 3121.7 ± 507.6 | 3238.2 ± 476.2 | <0.001 |
| Maternal age | 33.8 ± 3.6 | 33.3 ± 3.6 | 33.5 ± 4.4 | 0.632 |
| Maternal height(cm) | 159.5 ± 5.1 | 159.4 ± 8.8 | 160.4 ± 5.7 | 0.690 |
| Maternal weight before pregnancy (kg) | 51.7 ± 6.7 | 54.4 ± 8.2 | 59.5 ± 11.0 | <0.001 |
|  |  |  |  |  |
| Maternal weight during pregnancy (kg) ^b^ | 63.5 ± 7.0 | 66.6 ± 9.0 | 71.7 ± 11.8 | <0.001 |
|  |  |  |  |  |
| Gestational weight gain(kg) | 11.9 ± 3.3 | 12.2 ± 6.8 | 12 ± 15.5 | 0.94 |
| Maternal BMI before pregnancy | 20.3 ± 2.5 | 21.9 ± 8.7 | 23.2 ± 4.2 | 0.08 |
| Maternal BMI during pregnancy^b^ | 25 ± 2.5 | 26.8 ± 11.5 | 27.9 ± 4.2 | 0.196 |
| Paternal height(cm) | 174.5 ± 6.1 | 172.3 ± 5.2 | 173.8 ± 5.0 | 0.002 |
| Paternal weight | 72.8 ± 9.9 | 75.8 ±12.6 | 82 ± 13.2 | <0.001 |
| Paternal BMI | 23.9 ± 2.8 | 25.4 ± 3.6 | 27.4 ± 3.7 | <0.001 |
| Beverage (serving/day) | 0.15 ± 0.13 | 0.16 ± 0.16 | 0.2 ± 0.3 | 0.427 |
| Snack (serving/day) | 0.65 ± 0.4 | 0.75 ± 0.46 | 0.81 ± 0.4 | 0.127 |
| Exercise (hours/week) | 2.5 ± 4.1 | 2.1 ± 3.0 | 1.6 ± 2.1 | 0.260 |
| Maternal education at college or above (%) | 68 (82.9) | 290 (80.1) | 38 (66.7) | 0.044 |
| Paternal education at college or above (%) | 69 (84.2) | 292 (80.7) | 41 (71.9) | 0.192 |
| Family with higher income level(%) | 17 (20.7) | 94 (26.0) | 7 (12.3) | 0.062 |
| 1. Data were presented with the mean ± standard deviation or n (%) | | | | |
| 1. Maternal weight during pregnant is recorded from the latest routine prenatal visit | | | | |
| Abbreviation: BMI, body mass index ; NSD, normal spontaneous delivery. | | | | |
