# Supplementary material for: Association between maternal shift work during pregnancy child overweight and metabolic outcomes in early childhood
Source: Front Public Health. 2022 Sep 30;10:1006332. doi: 10.3389/fpubh.2022.1006332 (PMC9565036; doi:10.3389/fpubh.2022.1006332)
Supplement: Supplementary file 5 [file Table_5.docx]

| **Supplementary Table S5**. **Other demographic data between 2 groups** | | | | | | | |
| --- | --- | --- | --- | --- | --- | --- | --- |
|  |  | **Total population** | | | **Sub-population (Exclude underweight children)** | | |
|  | | Mother day work group (n=350) | Mother shift-work group (n=57) | p-value | Mother day work group (n=290) | Mother shift-work group (n=47) | p-value |
| Foreign spouse (%) | | 3 (0.9) ^a^ | 3 (5.3) | 0.151 | 3 (1) | 2 (4.3) | 0.294 |
| Maternal education level (%) | | 286 (81.7) | 45 (79) | 0.620 |  |  | 0.442 |
|  | Junior high | 0 (0) | 0 (0) |  | 0 (0) | 0 (0) |  |
|  | Senior high | 9 (2.6) | 4 (7.0) |  | 7 (2.4) | 3 (6.4) |  |
|  | Junior college | 55 (15.7) | 8 (14.0) |  | 47 (16.2) | 8 (17) |  |
|  | University | 187 (53.4) | 36 (63.2) |  | 151 (52) | 27 (57.5) |  |
|  | Graduate school | 99 (28.3) | 9 (15.8) |  | 85 (29.3) | 9 (19.2) |  |
| Paternal education level (%) | | 287 (82) | 43 (75.4) | 0.242 | 236 (81.4) | 34 (72.3) | 0.151 |
|  | Junior high | 1 (0.3) | 2 (3.5) |  | 1 (0.4) | 1(2.1%) |  |
|  | Senior high | 13 (3.7) | 4 (7.0) |  | 12 (4.2) | 4 (8.5) |  |
|  | Junior college | 48 (13.8) | 8 (14.0) |  | 40 (13.8) | 8 (17) |  |
|  | University | 146 (41.8) | 26 (45.6) |  | 118 (40.8) | 21 (44.7) |  |
|  | Graduate school | 141 (40.4) | 17 (29.8) |  | 118 (40.8) | 13 (27.7) |  |
| Maternal smoking (%) | | 0 | 1 (1.8) | 0.322 | 0 | 0 | NA |
| Maternal alcohol drinking (%) | | 9 (2.6) | 3 (5.3) | 0.389 | 7 (2.4) | 2 (4.3) | 0.556 |
| Maternal industry types | |  |  |  |  |  |  |
| Business | | 80 (23) | 11 (19.6) |  | 69 (24) | 8 (17.4) |  |
| Transportation | | 7 (2) | 0 |  | 6 (2) | 0 |  |
| Public administration | | 47 (13.5) | 2 (3.6) |  | 39 (13.5) | 1 (2.2) |  |
| Personal service | | 16 (4.6) | 7 (12.5) |  | 15 (5.2) | 7 (15.2) |  |
| Professional service | | 156 (44.8) | 31 (55.4) |  | 129 (44.8) | 28 (60.9) |  |
| Manufacture | | 25 (7.2) | 1 (1.8) |  | 16 (5.6) | 0 |  |
| Others | | 19 (5.4) | 5 (8.8) |  | 16 (5.6) | 3 (6.4) |  |
| Maternal lifting during work (%) | | 46 (18) | 17 (37.8) | *0.011 | 40 (17.9) | 15 (40.5) | *0.011 |
| Company employee > 30 (%) | | 94 (69.7) | 16 (55.2) | 0.135 | 84 (70.6) | 14 (56) | 0.157 |
| 1. Data were presented with n (%) | | | | | | | |
