# Supplementary material for: Association between maternal shift work during pregnancy child overweight and metabolic outcomes in early childhood
Source: Front Public Health. 2022 Sep 30;10:1006332. doi: 10.3389/fpubh.2022.1006332 (PMC9565036; doi:10.3389/fpubh.2022.1006332)
Supplement: Supplementary file 6 [file Table_6.docx]

|  | **Supplementary Table S6. Child beverage intake detail** | | | | | | | |
| --- | --- | --- | --- | --- | --- | --- | --- | --- |
|  |  |  |  |  |  |  |  |  |
|  |  |  | **Total population** | | | **Sub-population (Not include underweight children)** | | |
|  | | | Day work mothers group (n=350) | Shift-work mothers group (n=57) | p-value | Day work mothers group (n=290) | Shift-work mothers group (n=47) | p-value |
| **Handshake beverage** | | |  |  |  |  |  |  |
|  | Frequency | |  |  | 0.390 |  |  | 0.282 |
|  |  | ≧Once / day | 3 (0.9)^a^ | 0 |  | 3 (1.0) | 0 |  |
|  |  | ≧Once / week | 48 (13.7) | 9 (15.8) |  | 37 (12.8) | 7 (14.9) |  |
|  |  | ≧Once / month | 74 (21.1) | 9 (15.8) |  | 63 (21.7) | 7 (14.9) |  |
|  |  | < Once / month | 121 (34.6) | 22 (38.6) |  | 100 (34.5) | 20 (42.6) |  |
|  |  | never | 78 (22.3) | 9 (15.8) |  | 62 (21.4) | 6 (12.8) |  |
|  | Amount / once | |  |  | 0.876 |  |  | 0.663 |
|  |  | >750cc | 0 | 0 |  | 0 | 0 |  |
|  |  | 750cc | 0 | 0 |  | 0 | 0 |  |
|  |  | 500cc | 15 (4.3) | 2 (3.5) |  | 12 (4.1) | 2 (4.3) |  |
|  |  | 250cc | 62 (17.7) | 9 (17.8) |  | 57 (19.7) | 6 (12.8) |  |
|  |  | < 250cc | 167 (47.7) | 25 (43.9) |  | 130 (44.8) | 22 (46.8) |  |
| **Packaged beverage** | | |  |  |  |  |  |  |
|  | Frequency | |  |  | 0.406 |  |  | 0.719 |
|  |  | ≧Once / day | 7 (2.0) | 2 (3.5) |  | 6 (2.1) | 1 (2.1) |  |
|  |  | ≧Once / week | 88 (25.1) | 18 (31.6) |  | 71 (24.5) | 15 (31.9) |  |
|  |  | ≧Once / month | 102 (29.1) | 14 (24.6) |  | 82 (28.3) | 12 (25.5) |  |
|  |  | < Once / month | 79 (22.6) | 12 (21.1) |  | 70 (24.1) | 10 (21.3) |  |
|  |  | never | 42 (12.0) | 4 (7.0) |  | 32 (11.0) | 2 (4.3) |  |
|  | Amount / once | |  |  | 0.629 |  |  | 0.368 |
|  |  | >750cc | 0 | 0 |  | 0 | 0 |  |
|  |  | 750cc | 0 | 0 |  | 0 | 0 |  |
|  |  | 500cc | 16 (4.6) | 2 (3.5) |  | 14 (4.8) | 2 (4.3) |  |
|  |  | 250cc | 131 (37.4) | 21 (36.8) |  | 109 (37.6) | 14 (29.8) |  |
|  |  | < 250cc | 113 (32.3) | 20 (35.1) |  | 93 (32.1) | 19 (40.4) |  |
|  | 1. Data were presented with the mean ± standard deviation or n (%) | | | | | | | |
