# Supplementary material for: Association between maternal shift work during pregnancy child overweight and metabolic outcomes in early childhood
Source: Front Public Health. 2022 Sep 30;10:1006332. doi: 10.3389/fpubh.2022.1006332 (PMC9565036; doi:10.3389/fpubh.2022.1006332)
Supplement: Supplementary file 7 [file Table_7.docx]

|  | **Supplementary Table S7. Baseline characteristics of follow and non-follow up population** | | | |
| --- | --- | --- | --- | --- |
|  | | Follow group (n=501) | Non-followed group (n=529) | p-value |
| Parental factor | |  |  |  |
| Maternal age of pregnancy | | 33.4 ± 3.8 ^a^ | 33.5 ± 3.9 | 0.724 |
| Maternal height(cm) | | 159.9 ± 5.1 | 160.5 ± 5.4 | 0.051 |
| Maternal weight before pregnancy(kg) | | 54.5 ± 8.3 | 54.3 ± 8.0 | 0.654 |
| Maternal weight during pregnancy(kg) | | 66.8 ± 8.9 | 67 ± 9.4 | 0.690 |
| Gestational weight gain(kg) | | 12.2 ± 8.0 | 12.8 ± 6.1 | 0.192 |
| Maternal BMI before pregnant | | 21.8 ± 7.9 | 21.3 ± 5.6 | 0.219 |
| Maternal BMI during pregnant | | 26.1 ± 3.1 | 26 ± 3.3 | 0.646 |
| Paternal height(cm) | | 172.8 ± 5.5 | 173.1 ± 6.1 | 0.436 |
| Paternal weight (kg) | | 76.1 ± 12.6 | 75.2 ± 11.1 | 0.242 |
| Paternal BMI | | 25.4 ±3.6 | 25.1 ± 3.6 | 0.121 |
| Family high income level (%) | | 159 (33.7) | 214 (39.6) | 0.051 |
| Maternal high education level (%) | | 370 (78.4) | 424 (78.5) | 0.960 |
| Paternal high education level (%) | | 377 (79.9) | 427 (79.1) | 0.754 |
| Maternal industry (Professional service) | | 187 (39.6) | 194 (35.9) | 0.213 |
| Birth outcome | |  |  |  |
| Gestational age (weeks) | | 38.4 ± 3.1 | 38.2 ± 3.9 | 0.317 |
| Mode of delivery (NSD) | | 303 (64.2) | 313 (63.5) | 0.819 |
| Parity (First born) | | 274 (58.1) | 297 (60.2) | 0.884 |
| Birth Height(cm) | | 49.0 ± 2.8 | 48.9 ± 3.3 | 0.670 |
| Birth Weight(g) | | 3093.8 ± 530.5 | 3056.5 ± 504.4 | 0.262 |
| 1. Data were presented with the mean ± standard deviation or n (%)   Abbreviation: BMI, body mass index ; NSD, normal spontaneous delivery. | | | | |
